# RAF Kinase Inhibitor Protein in Myeloid Leukemogenesis

**DOI:** 10.3390/ijms20225756

**Published:** 2019-11-16

**Authors:** Armin Zebisch, Veronica Caraffini, Heinz Sill

**Affiliations:** 1 Division of Hematology, Medical University of Graz, 8036 Graz, Austria; veronica.caraffini@medunigraz.at (V.C.); heinz.sill@medunigraz.at (H.S.); 2 Otto Loewi Research Center for Vascular Biology, Immunology and Inflammation, Division of Pharmacology, Medical University of Graz, 8036 Graz, Austria

**Keywords:** RAF kinase inhibitor protein, RAS-signaling, hematopoiesis, myeloid neoplasias, acute myeloid leukemia

## Abstract

RAF kinase inhibitor protein (RKIP) is an essential regulator of intracellular signaling. A somatic loss of RKIP expression is a frequent event in solid human cancers, and a role of RKIP as metastasis-suppressor is widely accepted nowadays. Recently, RKIP loss has been described in acute myeloid leukemia (AML) and a series of other myeloid neoplasias (MNs). Functional in vitro and in vivo experiments revealed that RKIP is an essential player within the development of these liquid tumors; however, the respective role of RKIP seems to be complex and multi-faceted. In this review, we will summarize the current knowledge about RKIP in myeloid leukemogenesis. We will initially describe its involvement in physiologic hematopoiesis, and will then proceed to discuss its role in the development of AML and other MNs. Finally, we will discuss potential therapeutic implications arising thereof.

## 1. Introduction

RAF kinase inhibitor protein (RKIP; also known as phosphatidylethanolamine-binding protein 1 or PEBP1) is an essential regulator of intracellular signaling. In more detail, it modulates the signal propagation in many pivotal signal transduction cascades, including the RAS-mitogen activated protein kinase/extracellular signal-regulated kinase (MAPK/ERK), nuclear factor-κB (NF-κB), G-protein coupled receptor kinase-2 (GRK-2) and Glycogen synthase kinase 3β pathways [1,2,3,4,5,6,7]. Most studies focused on its role in the regulation of RAS-MAPK/ERK signaling [4]. This signaling module mainly mediates proliferative and anti-apoptotic signals from the cell surface to the respective intracellular effector proteins and is an essential player during malignant transformation [8,9,10,11]. It consists of the small G-proteins RAS (HRAS, KRAS, and NRAS), the three RAF isoforms ARAF, BRAF, and CRAF, as well as the dual-specificity kinases MEK 1/2 and ERK 1/2. Mechanistically, RKIP binds both CRAF and MEK. This prevents the physical interaction of these pathway members, and consequently, the propagation of the signal transduction [4].

In addition to its role as a physiologic regulator of intracellular signaling, RKIP has also been shown to be deregulated in a broad range of human malignancies. A somatic loss of RKIP expression was initially described in 2003 [12]. Fu et al. observed a decreased expression of RKIP in primary patient specimens of prostate cancer when compared to noncancerous prostate tissue. Even more interesting, RKIP expression levels were no longer detectable, when biopsies of prostate cancer metastases were studied. In agreement with these data, a complete or partial loss of RKIP expression has been described in a wide range of cancers nowadays [1,13,14,15]. RKIP loss thereby correlates with an aggravated disease course and the increased formation of metastases in the majority of cancer entities studied. These data suggest a role of RKIP as a metastasis-suppressor, which is also supported by functional in vitro and in vivo experiments, where RKIP decreased tumor cell invasion and migration [1,15,16].

Recently, RKIP loss has been described in acute myeloid leukemia (AML) and a series of other myeloid neoplasias (MNs). Functional in vitro and in vivo experiments revealed that RKIP is an important player within the development of these liquid tumors. However, the respective role of RKIP seems to be complex and multi-faceted. In this review, we will summarize the current knowledge about the role of RKIP in physiologic and malignant myeloid hematopoiesis, with a specific focus on AML. Moreover, we will discuss potential therapeutic implications arising thereof.

## 2. RKIP in Physiologic Hematopoiesis

To discuss the role of RKIP in myeloid leukemogenesis, we initially have to address its relevance in physiologic hematopoiesis. Principally, hematopoiesis arises from hematopoietic stem cells (HSCs), which have the potential for self-renewal on the one hand, and differentiation into all types of blood cells on the other hand. While the complete and complex system of hematopoietic differentiation is reviewed in detail elsewhere [17,18,19], this review will focus on those steps, where the relevance of RKIP has been shown. As a general principle, HSCs can differentiate into the lymphoid and the myeloid lineage. The lymphoid lineage comprises all lymphatic cells, including B- and T-lymphocytes, as well as Natural Killer (NK) and lymphoid dendritic cells. The myeloid lineage contains the granulocytic cells, including basophil, eosinophil, and neutrophil granulocytes on the one hand, as well as the monocyte/macrophage cell compartment on the other hand. Additionally, myelopoiesis includes thrombopoiesis, erythropoiesis, as well as the formation of mast cells. While older models of hematopoiesis have proposed a strict separation between the myeloid and lymphoid branches already at the earliest steps of HSC differentiation, this picture turned out to be more complex in recent times. It is now widely accepted that the myeloid and lymphoid lineage remain connected further down in the differentiation model until the stage of lymphoid-primed multipotential progenitors (LMPP), which have the potential to develop into both the common lymphoid progenitor (CLP) and granulocyte–monocyte progenitor (GMP) compartments. Additionally, the GMP population has been demonstrated to be fairly heterogeneous and might develop into dendritic cells (DCs) as well. [20]. 

A first insight into the role of RKIP in hematopoiesis stems from expression analyses in various hematopoietic cell compartments. Several studies, including work from our laboratory, have shown that RKIP is prominently expressed in CD34^+^ hematopoietic stem and progenitor cells (HSPCs). However, its expression is greatly decreased in specimens from whole blood, which contain mainly mature and differentiated blood cells [1,21,22,23]. More detailed analyses revealed that this phenomenon is caused by a significantly decreased RKIP expression in differentiated myeloid leukocytes, including granulocytes and monocytes. On the contrary, RKIP levels remain high in lymphocytes, which show an expression comparable to the HSPC compartment [1,21,22]. This observation was also corroborated in murine in vivo models. Again, *Rkip* expression was high in HSPCs and lymphoid cells, but significantly decreased in mature myeloid cells, including monocytes and granulocytes [21,24]. A more detailed look at specific stages of myeloid differentiation demonstrated that *Rkip* levels remain high during the early stages of myelopoiesis. These include the common myeloid progenitor (CMP) and GMP stages, where *Rkip* expression is similar to the immature lin^−^/Sca^+^/kit^+^ (LSK) HSPC compartment. Subsequently, *Rkip* decreases during the terminal stages of granulo-monocytic differentiation [21,24,25,26,27]. These data suggest a functional involvement of RKIP in myelomonocytic differentiation. This hypothesis is further driven by the fact that RAS-MAPK/ERK activation is relevant for the myelomonocytic lineage commitment of HSPCs. This has been shown previously by Wang et al., who studied the phosphorylation of ERK during myelomonocytic differentiation of HL-60 cells [28]. HL-60 is an undifferentiated AML cell line that can be differentiated into granulocytes, monocytes or macrophages by the addition of either all-trans-retinoic acid (ATRA), Vitamin D3, or phorbol 12-myristate 13-acetate (PMA), respectively [29]. The authors observed that ERK phosphorylation was significantly increased during this differentiation process, and that the differentiation could be inhibited by incubation with a pharmacological inhibitor of MEK. In line with these data, modulation of RAS-MAPK/ERK regulators, including AKT, Kinase suppressor of RAS (KSR), Kinase suppressor of RAS-2 (KSR-2), and Cobalt uptake protein1 (COT1), influences the myelomonocytic differentiation of HL-60 cells as well [30,31,32,33]. To study the role of RKIP during the myelomonocytic differentiation process, we used primary human CD34^+^ HSPCs and performed a knockdown of RKIP by lentiviral transduction. Subsequently, we induced myelomonocytic differentiation by a mix of hematopoietic growth factors, including granulocyte-macrophage colony-stimulating factor (GM-CSF). RKIP knockdown greatly induced the expression of the myelomonocytic surface markers CD11b and CD14, which indicates that RKIP knockdown increases the myelomonocytic differentiation of HSPCs. Interestingly, RKIP knockdown without additional GM-CSF stimulation was insufficient to induce the differentiation process. These data could be corroborated in the above-mentioned HL-60 model. Again, RKIP knockdown increased the Vitamin D3 induced myelomonocytic differentiation, whereas RKIP overexpression inhibited this process. Once more, RKIP knockdown without Vitamin D3 incubation failed to induce HL-60 differentiation. Ultimately, these data were also confirmed in an in vivo setting employing a murine model with a complete knockout of the *Rkip* gene. As seen in the in vitro experiments, *Rkip* deletion in these mice increased the GM-CSF induced myelomonocytic differentiation, resulting in increased levels of granulocytes and monocytes in the peritoneal cavity, the peripheral blood, and the bone marrow. As described for the in vitro assays, *Rkip* deletion alone—without GM-CSF administration—was insufficient to induce myelomonocytic differentiation [21]. This suggests that RKIP downregulation acts as an amplifier in myelomonocytic differentiation, but that it is insufficient to induce the process on its own. The same pattern has been described for other RAS-MAPK/ERK regulators previously [32].

Another interesting observation comes from Schuierer et al., who observed that the decreased expression of RKIP in monocytes starts to rise again when they transform into macrophages [23]. In functional studies, the authors were able to show that RKIP is indeed functionally involved in this process, as its overexpression in the monocytic AML cell line THP-1 caused the differentiation of these cells into macrophages.

**Figure 1:**
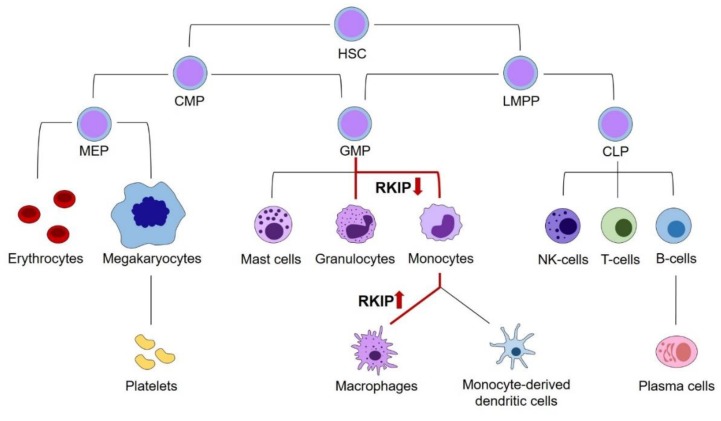
The role of RKIP in physiologic hematopoiesis. Schematic presentation of hematopoiesis. A decrease of RKIP expression occurs at the GMP stage and is an important step in monocyte and granulocyte differentiation. However, in a physiologic setting, the decreased expression in these cells is not irreversible and resurgence seems to be an essential step in the terminal differentiation of monocytes into macrophages. Changes in RKIP expression are highlighted by red arrows. HSC, hematopoietic stem cells; CMP, common myeloid progenitors; LMPP, lymphoid-primed multipotential progenitors; CLP, common lymphoid progenitors; GMP, granulocyte–monocyte progenitors; MEP, megakaryocyte–erythrocyte progenitors.

These data create an interesting picture, where a decrease of RKIP expression in HSPCs is an important step for their commitment into the myelomonocytic lineage and contributes to the formation of monocytes and granulocytes (Figure 1). However, in a physiologic setting, the decreased expression of RKIP in these cells is not irreversible, and a resurgence seems to be an essential step in the terminal differentiation of monocytes into macrophages. On the contrary, RKIP expression levels seem to be unaffected during the lymphoid differentiation [1,21,22,23].

## 3. RKIP in AML and Other Myeloid Neoplasias

AML is an aggressive hematopoietic malignancy that arises from the malignant transformation of HSPCs. Despite the use of intensive treatment strategies, including hematopoietic stem cell transplantation for younger and/or medically fit patients, and the establishment of several non-intensive treatment options for older individuals, the prognosis of AML is still dismal [34,35,36,37,38,39,40]. The cure rates range from 35 to 40% in patients younger than 60 years, and only from 5 to 15% in patients who are older than 60 years [40,41]. 

Pathogenetically, almost all AML cases exhibit recurrent genetic aberrations. These include mutational and non-mutational events, the latter comprising gene fusions, copy-number alterations, aberrant methylation signatures, as well as pathologic gene and non-coding RNA expression profiles. [42,43,44,45,46]. Importantly, however, AML is a genetically heterogeneous disease and molecular aberrations with functional relevance for AML development could be detected in a broad range of genes [9,40,45,46]. Moreover, AML pathogenesis is a multistep process, which is characterized by the continuing acquisition of (epi-)genetic aberrations [47,48]. This process often commences in HSCs long before the development of frank AML. In detail, HSCs acquire specific aberrations, thereby transforming into so-called pre-leukemic stem cells. While these cells already harbor some key features of leukemogenesis, they are still able to differentiate into all hematopoietic lineages and fail to establish AML. Through the continuing acquisition of additional aberrations, they finally transform into leukemic stem cells (LSCs) and thereby cause the establishment of a full-blown AML [47,49,50]. This model implies that some driver mutations are relevant from the stage of pre-leukemic stem cells until the phase of frank AML development and maintenance, as shown for mutations in *TP53* [51,52,53]. However, this model also highlights the fact that the continuing acquisition of mutations produces a situation in which genetic aberrations usually do not exist as solitary events. Indeed, a multitude of (epi-)genetic variations can usually be detected in every AML patient at the same time [46]. Importantly, they have also been shown to interact with each other and to drive myeloid leukemogenesis synergistically in many cases.

The significance of RKIP for AML has first been highlighted by the observation of an RKIP expression loss in two patients with therapy-related AML (t-AML) [54]. RKIP loss thereby described a somatic and leukemia-specific aberration, which promoted the malignant transformation in vitro. Most interestingly, however, previous work demonstrated that these patients additionally exhibited a germline mutation within the CRAF oncogene [55]. These mutations were shown to be oncogenic and activated RAS-MAPK/ERK signaling in in vitro experiments. However, despite their germline origin, constitutive activation of the RAS-MAPK/ERK pathway was only observed in malignant tissues of these patients but not in the surrounding normal cells. These data suggest that the interaction with additional (epi-)genetic aberrations is necessary to unravel the full leukemic potential of mutated CRAF. Indeed, functional studies revealed that CRAF mutations and RKIP loss synergized in the activation of RAS-signaling on the one hand, and oncogenic transformation on the other hand [54]. Taken together, these results indicate that RKIP loss is a leukemia-specific, secondary genetic aberration in patients with CRAF germline mutations and contributes to a CRAF-induced leukemic transformation within these cases.

Importantly, a loss of RKIP cannot only be observed in the small subgroup of t-AML with CRAF germline mutations but seems to be a more general phenomenon in AML. By studying more than 400 primary patient specimens derived from patients with all different subtypes of AML, we described a loss of RKIP as one of the most frequent molecular aberrations occurring in AML, affecting more than 20% of patients [56]. We could also demonstrate that RKIP inhibits the proliferation and clonogenic growth of a series of AML cell lines, which suggests a potential tumor-suppressor role within this neoplasia [56,57]. Moreover, we could demonstrate that RKIP indeed regulates the RAS-MAPK/ERK pathway in these cells, which suggests that the anti-tumor effects of RKIP in myelopoiesis are mediated via the inhibition of this signaling cascade. Of note, the aberrant RKIP function in AML seems to be mediated by a loss of RKIP expression only. This is demonstrated by the fact that mutations and/or copy number alterations have not been detected in primary AML patient specimens thus far [54,56]. While these data suggest a tumor-suppressor role of RKIP in AML, this is in discordance with the fact that RKIP primarily acts as a metastasis-suppressor in solid cancers. In this respect, it is worth to mention that AML is a systemic disease. Consequently, metastases do not exist. However, it is interesting to look at the subgroup of myeloid sarcoma (MS) in this respect. In this situation, AML blasts form solid tumor masses in non-hematopoietic tissues, a condition that can even precede the occurrence of AML within the bone marrow [58,59,60,61]. Therefore, leukemic blasts have to invade into and migrate within extramedullary tissues, a process resembling the formation of metastases in solid malignancies. We, therefore, asked for the role of RKIP in MS and studied its expression in AML patients with and without extramedullary manifestations. Indeed, RKIP loss was frequently observed in MS biopsies [62]. Interestingly, loss of RKIP at the biopsy site correlated with RKIP loss in the corresponding bone marrow specimen of MS patients. This is of interest for clinical application, as RKIP expression in the bone marrow can easily be studied during a routine diagnostic work-up of AML. Consequently, RKIP expression in the bone marrow might be used as a biomarker with the potential to predict the occurrence of extramedullary AML disease. On a functional level, RKIP depletion increased the invasion and migration potential of a series of AML cell lines. Importantly, these findings could also be corroborated in vivo, as RKIP knockdown increased the potential of leukemic cells to invade the chorioallantoic membrane of chicken embryos, and to form solid tumor masses therein [62]. Taken together, these results demonstrate that the metastasis-suppressor function of RKIP is also relevant for AML, and that it contributes to the formation of extramedullary manifestations within this disease.

**Figure 2:**
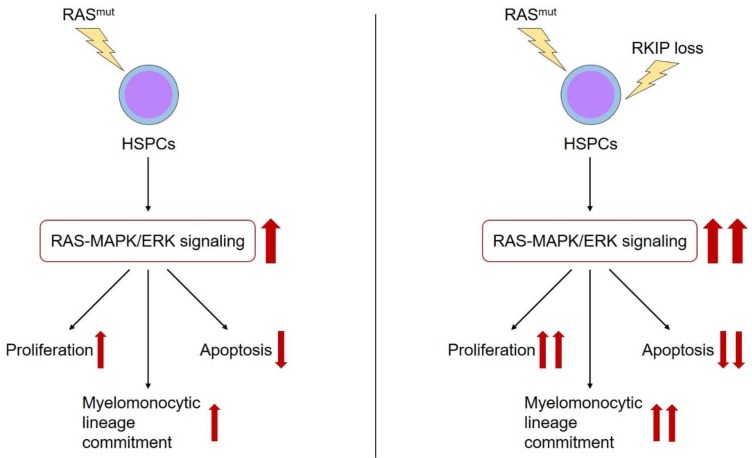
The role of RKIP in myeloid leukemogenesis. RAS-signaling mutations (RAS ^mut^) are essential genetic aberrations during the leukemic transformation of HSPCs. Among other effects, they activate the RAS-MAPK/ERK signaling cascade, which in turn i) increases the proliferation, ii) reduces the apoptosis, and iii) induces the myelomonocytic lineage commitment of HSPCs (red arrows). Additional occurrence of RKIP loss has been shown to aggravate the effects of RAS-signaling mutations on these properties (red arrows).

As outlined above, RKIP is a negative regulator of RAS-MAPK/ERK signaling. Therefore, it is worth to focus on the interaction between RKIP and other alterations within the RAS-MAPK/ERK pathway in more detail. As mentioned above, RKIP loss correlated with CRAF germline mutations in patients with t-AML. As these alterations also synergized in malignant transformation, one might hypothesize that RKIP loss describes a secondary genetic event in patients with RAS-signaling mutations. In this situation, RKIP would amplify the activating properties of these aberrations and thereby potentiate their oncogenic effects (Figure 2). In agreement with this hypothesis, we could demonstrate that RKIP loss correlates with RAS-signaling mutations in AML. This is true for the subgroup of MS, but also in a larger cohort of almost 400 AML patients comprising all different AML subgroups [56]. Indeed, functional experiments revealed that the overexpression of RKIP inhibited the oncogenic potential of mutated RAS, which further supports the hypothesis mentioned above of synergistic effects between RAS-signaling mutations and RKIP loss. 

Finally, it is worth to look at the role of RKIP in other myeloid neoplasias (MNs) as well. This is mainly because some general mechanisms of myeloid leukemogenesis are not specific to AML, but also apply to other MN subtypes. In return, studying the mechanisms of RKIP loss in these diseases might also help to learn more about mechanisms that might apply to AML development. Li et al. described a loss of RKIP in chronic myeloid leukemia (CML) [63]. In agreement with the results from AML, RKIP inhibited the proliferation, viability, and clonogenic growth of CML cells. Again, this correlated with the inhibition of RAS-MAPK/ERK signaling. Another interesting MN subtype for studying RKIP expression is chronic myelomonocytic leukemia (CMML). CMML is an aggressive malignancy of the HSCs, where the malignant offspring is characterized by both increased proliferation on the one hand, and increased myelomonocytic differentiation on the other hand [64,65,66]. Additionally, CMML exhibits an inherent risk of AML transformation. A possible role for RKIP within this disease is based on the observation mentioned above that decreased RKIP expression levels induce not only the proliferation and tissue invasion of hematopoietic cells but also the myelomonocytic lineage commitment of HSPCs. In our lab team, we could show that RKIP loss occurs in almost 30% of CMML cases and that RKIP depletion contributed to CMML pathogenesis in functional in vitro and murine in vivo models, respectively [21]. In line with the data presented above, RKIP loss correlated with RAS-signaling mutations and increased the effects of these mutations on activation of the RAS-MAPK/ERK cascade. As *Rkip* deletion even aggravated the CMML development in *Nras^G12D^* mice,[21]. these data again highlight the relevance of the interaction between RAS-signaling mutations and RKIP loss in myeloid leukemogenesis.

## 4. RKIP as a Therapeutic Target in AML and Other MNs

A complete or partial loss of RKIP expression is frequently described in MNs, and functional relevance of this RKIP loss for myeloid leukemogenesis could be proven in vitro and in vivo by other groups and us [21,56,63]. Therefore, re-expression of RKIP represents an interesting therapeutic approach in AML and other MNs. To achieve this goal, the mechanisms behind the development of RKIP loss have to be deciphered first. Studies in large cohorts of primary AML patient specimens excluded the occurrence of RKIP mutations, copy number deletions, as well as the hypermethylation of the RKIP promoter region [54,56,67]. Particularly, the absence of hypermethylation within the RKIP promoter is of therapeutic relevance, as this suggests that demethylating agents, including azacitidine and decitabine, do not display a promising therapeutic approach to restore RKIP expression. These data are in contrast to results from several solid tumors, where hypermethylation of RKIP has been demonstrated [68,69,70], and that show that the reasons for RKIP loss might be tissue-specific. To shed more light on the reasons for RKIP loss in AML, we screened for the deregulation of micro-RNAs (miRs). miRs are small, non-coding RNA fragments, which play a central role in the regulation of cellular gene expression profiles [71]. Aberrant expression of miRs frequently occurs in human malignancies and is involved in the process of malignant transformation [42,72,73,74,75]. Recently, a series of miRs have been shown to target and downregulate RKIP in solid cancers, including miR-224, miR-27a, and miR-543 [76,77,78]. By studying more than 400 AML patient specimens, we observed an increased expression of miR-23a within this disease and demonstrated that the miR-23a levels correlated inversely with the expression of RKIP. We could further show that miR-23a directly targets the 3′UTR of RKIP, which in turn causes RKIP downregulation in hematopoietic cells [57]. In functional experiments, miR-23a overexpression mimicked the effects of RKIP knockdown, as it induced the proliferation of AML cell lines. Moreover, the effects of miR-23a overexpression could thereby be rescued by the simultaneous expression of an RKIP construct with a defective miR-23a binding site. These experiments prove the existence of a functionally relevant miR-23a/RKIP axis in AML and describe miR-23a as a potential therapeutic target to overcome the effects of RKIP loss within this malignancy. Such an outlook is further fueled by the fact that the modulation of miR expression profiles in humans is technically feasible, and that the development of miR-therapeutics is already well advanced [79]. When discussing the reasons behind RKIP loss in MNs, it is worth to notice that the function of RKIP as an inhibitor of RAS-MAPK/ERK signaling cannot only be inactivated by a loss of expression, but also by increased phosphorylation (p-Ser153 RKIP). This phosphorylation is caused by protein kinase C (PKC) [6,80] and often accompanied by a paradoxically increased expression of RKIP [81]. Mechanistically, p-Ser153 RKIP has lost its ability to interact with CRAF, which in turn impedes its inhibitory function on RAS-MAPK/ERK signaling. This is of interest, as the levels of p-Ser153 RKIP have been shown to be relevant for several solid tumors and lymphoid neoplasias, and as a correlation with poor prognosis was reported in many of these entities [1,81,82,83,84,85]. Furthermore, phosphorylation of RKIP is of interest from a therapeutic point of view as well, as pharmacologic inhibition of PKC was able to inhibit the phosphorylation of RKIP [6,80]. Despite all these data, it is currently unknown whether phosphorylation of RKIP occurs in AML and/or other MNs. Future studies will have to shed more light on this issue.

Another possibility to use RKIP for optimizing the treatment of AML is not to modulate the expression of RKIP, but rather to use it as a biomarker for the sensitivity to currently used cytotoxic agents. We have previously shown that decreased expression of RKIP correlated with superior survival in intensively treated AML patients. These data are in line with reports of increased sensitivity to cytarabine in AML patients with activated RAS [86,87,88,89] and suggest an involvement of the RAS-MAPK pathway in the sensitivity to cytarabine. This is particularly relevant for older patients, where the use of high-intensity chemotherapeutic approaches is still a matter of debate, and where other, non-intensive therapeutic approaches, such as hypomethylating agents and low-dose cytarabine, are already available [34,35,36,37,38,39,40]. One might speculate that RKIP expression levels might be additionally used to decide whether older patients are still treated with high-dose chemotherapy or whether they are better switched to a non-intensive alternative upfront. As a limitation, it has to be noted that the clinical data about the prognostic role of RKIP in AML should be confirmed in larger and prospective cohorts first. Moreover, it is currently unknown whether RKIP expression might play a role as a predictive marker for non-intensive therapeutic regimens as well. Another possibility for the potential use of RKIP in optimizing therapeutic approaches stems from the observation that RKIP loss co-occurs with RAS-signaling mutations in MNs, including AML, and that this co-occurrence has been shown to hyperactivate the RAS-MAPK pathway in in vitro and in vivo models [21,56,62]. A similar finding has been shown for the co-occurrence of RAS and TET2 mutations [90]. In this situation, mutant TET2 caused a downregulation of the RAS-signaling inhibitor SPRY2, which in turn synergized with the RAS mutation in hyperactivation of the RAS-MAPK signaling cascade. Importantly, however, the authors demonstrated that this RAS-MAPK hyperactivation increased the dependency on RAS-signaling within affected MNs. Consequently, this co-occurrence caused a particular sensitivity to MAPK inhibitors, including the MEK inhibitor binimetinib. It will be interesting to delineate whether these findings also apply to the co-occurrence of RAS-signaling mutations and RKIP loss, and whether this might reveal a novel possibility to identify patients, who will profit from treatment with MAPK-signaling inhibitors.

## 5. Conclusions

RKIP plays a major role in physiologic hematopoiesis and myeloid malignancies. In physiologic hematopoiesis, a decrease of RKIP expression in the HSPC pool increases the myelomonocytic lineage commitment of these cells. Importantly, low expression levels of RKIP have been detected in AML and a variety of other MNs, and a functional involvement in myeloid leukemogenesis has been proven. Finally, RKIP expression is of prognostic relevance and might display an exciting candidate for optimizing therapeutic strategies in AML.
